# Selective oxidation of alcohol-*d*_1_ to aldehyde-*d*_1_ using MnO_2_†

**DOI:** 10.1039/d1ra05405h

**Published:** 2021-08-24

**Authors:** Hironori Okamura, Yoko Yasuno, Atsushi Nakayama, Katsushi Kumadaki, Kohei Kitsuwa, Keita Ozawa, Yusaku Tamura, Yuki Yamamoto, Tetsuro Shinada

**Affiliations:** Graduate School of Science, Osaka City University Sugimoto, Sumiyoshi Osaka 558-8585 Japan shinada@sci.osaka-cu.ac.jp

## Abstract

The selective oxidation of alcohol-*d*_1_ to prepare aldehyde-*d*_1_ was newly developed by means of NaBD_4_ reduction/activated MnO_2_ oxidation. Various aldehyde-*d*_1_ derivatives including aromatic and unsaturated aldehyde-*d*_1_ can be prepared with a high deuterium incorporation ratio (up to 98% D). Halogens (chloride, bromide, and iodide), alkene, alkyne, ester, nitro, and cyano groups in the substrates are tolerated under the mild conditions.

## Abstract 2

A facile method for deutrium incorporation into aldehydes by mild reduction of NaBD_4_ of aldehydes and MnO_2_ oxidation (98% D) is disclosed.

## Graphical abstract

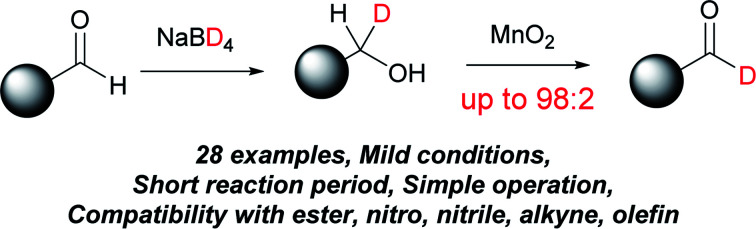

## 1. Introduction

Deuterium (^2^H, d) is a stable, non-radioactive, and safe isotope of hydrogen (^1^H). Since its discovery,^1^ d has been widely utilized in organic chemistry, biochemistry, analytical chemistry, pharmaceutical science, and drug discovery.^2,3^ Because of the high demand for d-labelled molecules in the scientific research fields, many efforts have been devoted to developing a new method for the synthesis of d-labelled molecules.

Aldehyde-*d*_1_2 has received significant attention as a synthetic target due to the facts that aldehyde 1 is a useful feedstock in organic synthesis. Various methods have been performed in the synthesis of alkyl and aryl aldehyde-*d*_1_. For example, more than 40 syntheses (25 different reaction conditions) of benzaldehyde-*d*_1_ (PhCDO) were conducted even since 2018 in the studies to develop new d-incorporation method or reaction mechanism using PhCDO.^4–8^

**Scheme 1:**
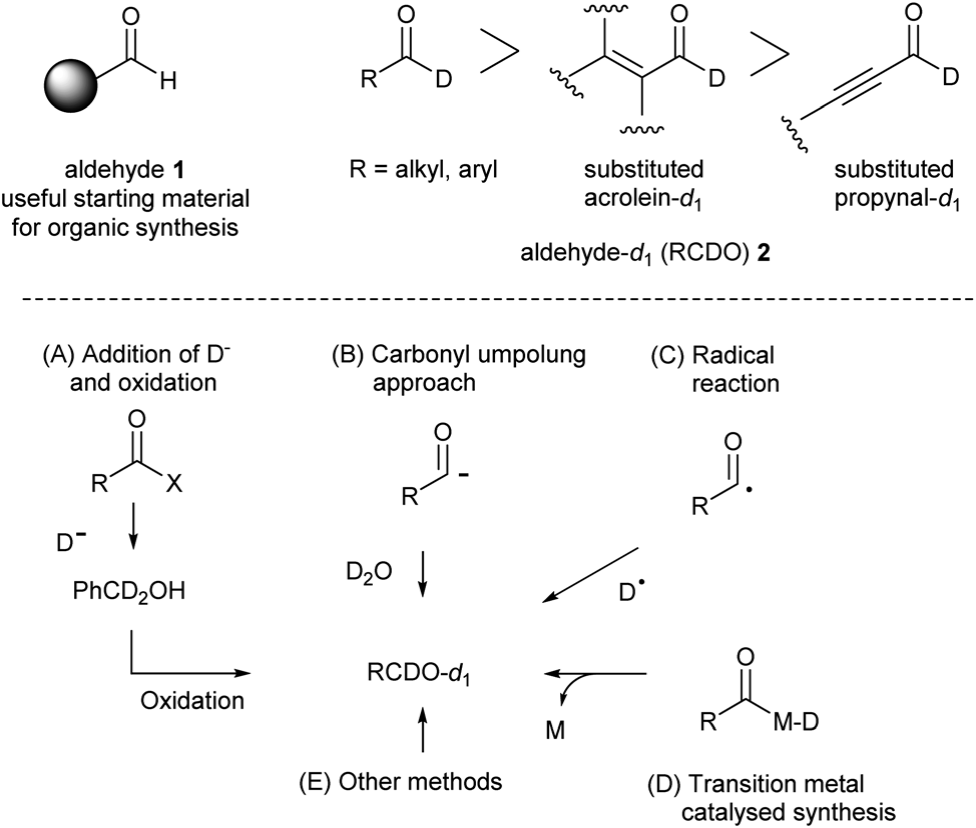
Synthesis of aldehyde-*d*_1_ derivatives. (A) reduction of the formyl with D^−^ and oxidation, (B) carbonyl Umpolung approach, (C) radical H–D exchange, (D) transition metal-catalysed H–D exchange, and (E) others.

The previous synthetic approaches to access d-labelled molecules are classified into 5 types; (A) addition of D^−^ followed by oxidation, (B) carbonyl Umpolung approach, (C) radical reaction, (D) transition metal-catalysed reaction, and (E) others. Recently, mild, one-step, and catalytic syntheses of aldehyde-*d*_1_2 have been achieved by deuteration of the Breslow intermediates,^9^ deuteration of acyl radicals,^6,10^ and transition metal-catalysed deuterium incorporation.^11^ However, the previous synthetic methods including the modern direct syntheses often suffered from drawbacks such as over-deuteration, requirements of harsh conditions (high and low temperature, and strong base and acids), and the use of expensive catalysts. Moreover, the synthetic examples of substituted acrolein and propynal-*d*_1_ are much less than those of alkyl and aryl aldehyde-*d*_1,_^12,13^ though recently developed NHC-catalysed H–D exchange reactions allowed access to various substituted acrolein-*d*_1_ derivatives.^9^ In this context, development of a new d-incorporation method which allows flexible synthesis of aromatic and unsaturated aldehyde-*d*_1_2 remains to be a challenging synthetic task (Scheme 1).

**Scheme 2:**
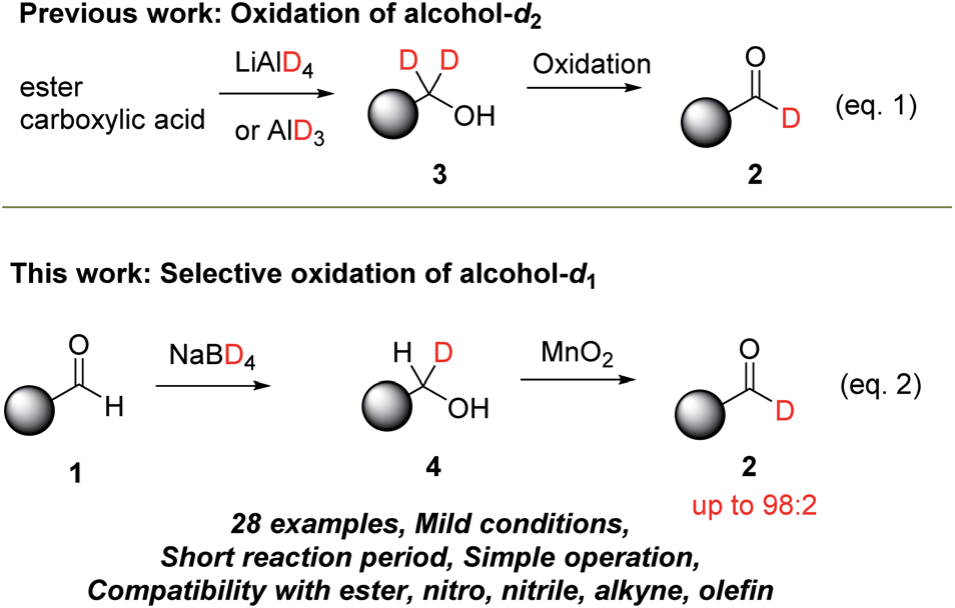
Oxidation of deuterated alcohols. Eqn (1): oxidation of alcohol-*d*_2_, eqn (2): selective oxidation of alcohol-*d*_1_.

Method A using D^−^ as a deuterium source has been recognized as a robust and conventional synthetic method to prepare aldehyde-*d*_1_2 (Scheme 2). The synthesis is typically performed in two steps; (i) reduction of carboxylic acid derivatives using LiAlD_4_ to provide alcohol-*d*_2_3 and (ii) oxidation to aldehyde-*d*_1_2 (eqn (1)).^14^ In this approach, the deuterium incorporation ratio (%D) of the commercially available D^−^ sources such as LiAlD_4_ (>98 atom) is reliably transferred into the product. On the other hand, the use of highly reactive LiAlD_4_ often limits the synthetic scope. Under the conditions, various functional groups such as nitro, nitrile, ester, and acid moieties, and alkene and alkyne with electron-withdrawing group(s) are not tolerated. To overcome the limitation, we emerged selective oxidation of alcohol-*d*_1_ derivatives 4 (Scheme 2(eqn (2))). It is expected that various alcohol-*d*_1_4 can be prepared by the mild NaBD_4_ reduction. The next selective oxidation of D (H/D selectivity) is the key to this approach. Recently, oxidation of benzyl alcohol-*d*_1_ (PhCDHOH) with PCC or PDC was conducted to prepare PhCDO with ∼85%D.^4*a*,4*e*,4*q*^ On the other hand, further efforts to improve the selectivity (%D) in the selective oxidation have not been well-examined. Herein, we would like to report that NaBD_4_ reduction followed by activated MnO_2_ oxidation (NaBD_4_/MnO_2_ system). The simple and mild protocol allows expansion of the synthetic range of aldehyde-*d*_1_2 including not only aromatic aldehyde-*d*_1_ derivatives but also substituted acrolein-*d*_1_ and propynal-*d*_1_ derivatives with high %D (up to 98%).

## 2. Results and discussion

**Scheme 3:**
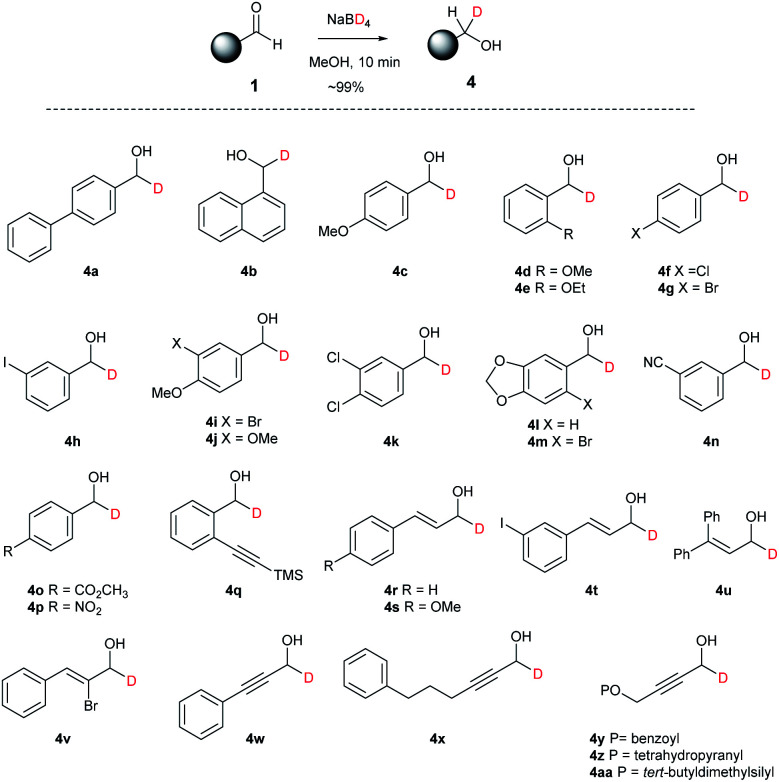
Reduction of aldehyde 1 with NaBD_4_.

In a similar manner to the previous synthetic examples of NaBH_4_ reduction of aldehyde 1, the reduction with NaBD_4_ gave the corresponding alcohol-*d*_1_ derivatives 4 with excellent functional group compatibility and yields (Scheme 3). Chloride, bromide, iodide, methoxy, ethoxy, or methylene acetal, nitrile, ester, nitro, and alkyne groups on the aromatic ring of 4c–4q were tolerated under the conditions. Substituted acrolein and propynal 1r–1aa also underwent smooth NaBD_4_ reduction to provide 4r–4aa without loss of the alkyne and alkene moieties, and tetrahydropyanyl (THP), benzoyl (Bz), and *tert*-butyldimethylsilyl (TBS) protecting groups.

We next examined the key oxidation of alcohol-*d*_1_4 using 4-phenylbenzyl alcohol-*d*_1_ (4a) (Table 1). As a result, activated MnO_2_ was found to be superior to other general oxidation reagents (entry 1, Table 1). Treatment of 4a with 23 eq. of MnO_2_ in CH_2_Cl_2_ gave aldehyde-*d*_1_2a with 92% D in 2 h. The use of pyridinium dichlorochromate (PDC) gave 2a in good selectivity (88% D).^4*e*^ However, the isolate yield was moderate (entry 2). Dess–Martin periodinane oxidation, 2,2,6,6-tetramethylpiperidine 1-oxyl (TEMPO) oxidation in the presence of PhI(OAc)_2_, and Parikh–Doering oxidation (sulfur trioxide–pyridine complex in dimethyl sulfoxide (DMSO)) resulted in lower selectivities (74, 76 and 66%D, entries 3–5).

**Table 1:** Oxidation of alcohol-*d*_1_4a^a^

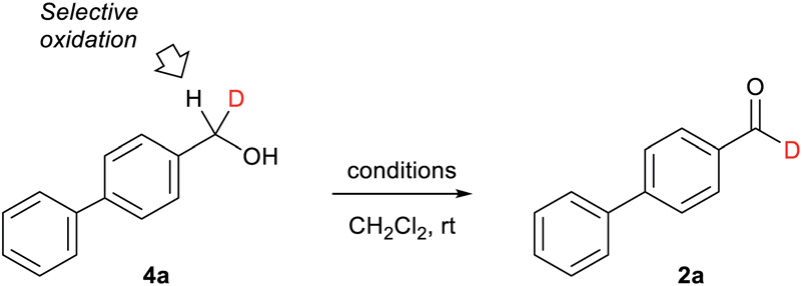			
Entry	Conditions	Yield^b^ (%)	%D^c^
1	MnO_2_ (23 eq.), 1 h	92	92
2	PDC (1.2 eq.), MS4A, 2 h	51	88
3	Dess–Martin periodinane (1.5 eq.), 5 min	84	74
4	TEMPO (0.01 eq.), Bu_4_NHSO_4_ (0.05 eq.), NaOCl (1.2 eq.), 1 h	96	76
5	DMSO (10 eq.), SO_3_–pyridine (4 eq.), *i*Pr_2_NEt (5 eq.), 1.5 h	75	66

a 0.5 mmol scale.

b Isolated yield.

c %D for 2a is calculated based on the integration ratios of aldehyde and aromatic proton. MnO_2_ = activated manganese dioxide, PDC = pyridinium dichlorochromate, MS4A = molecular sieves 4A, TEMPO = 2,2,6,6-tetramethylpiperidine 1-oxyl, DMSO = dimethyl sulfoxide.

**Scheme 4:**
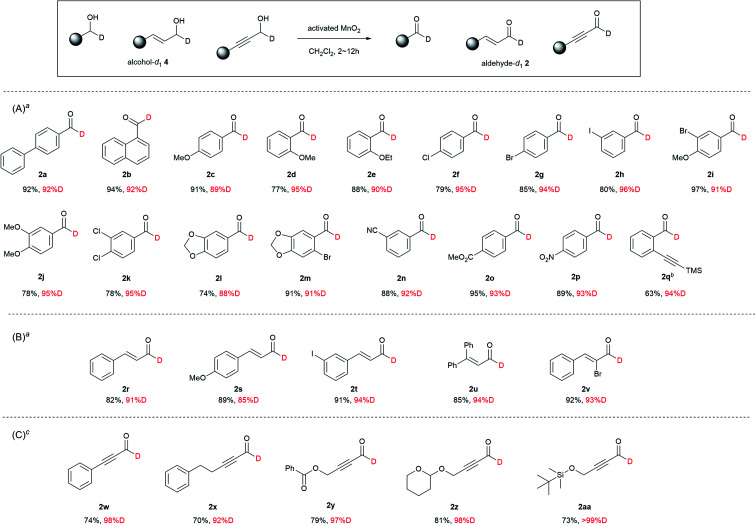
MnO_2_ oxidation of alcohol-*d*_1_4. (A) synthetic examples of aromatic aldehyde-*d*_1_2, (B) synthetic examples of substituted acrolein-*d*_1_2, and (C) synthetic examples of substituted propynal-*d*_1_2. ^*a*^2 h, ^*b*^6 h, ^*c*^12 h.

Activated MnO_2_ oxidation was successfully expanded to the synthesis of various aldehyde-*d*_1_2a–2aa with high %D (85–96%D) (Scheme 4A–C). Chloride, bromide, iodide, methoxy, ethoxy, or methylene acetal, nitrile, ester, nitro, and alkyne groups on the aromatic ring of 4c–4q are preserved under the mild oxidation conditions (Scheme 4A). Substituted acrolein 4r–4v and propynal 4w–4aa smoothly underwent MnO_2_ oxidation to provide 2r–2aa without loss of the alkene and alkyne moieties (Scheme 4B and C). The synthetic utility was further demonstrated by the synthesis of 2v with a bromo group at the α-position of cinnamaldehyde. In addition, Bz, THP, and TBS protecting groups of 4y, 4z, and 4aa were also maintained under the conditions. These propargyl alcohols 4y, 4z, and 4aa were smoothly converted to the corresponding propynal derivatives 2y, 2z, and 2aa with high %D, respectively.

**Scheme 5:**
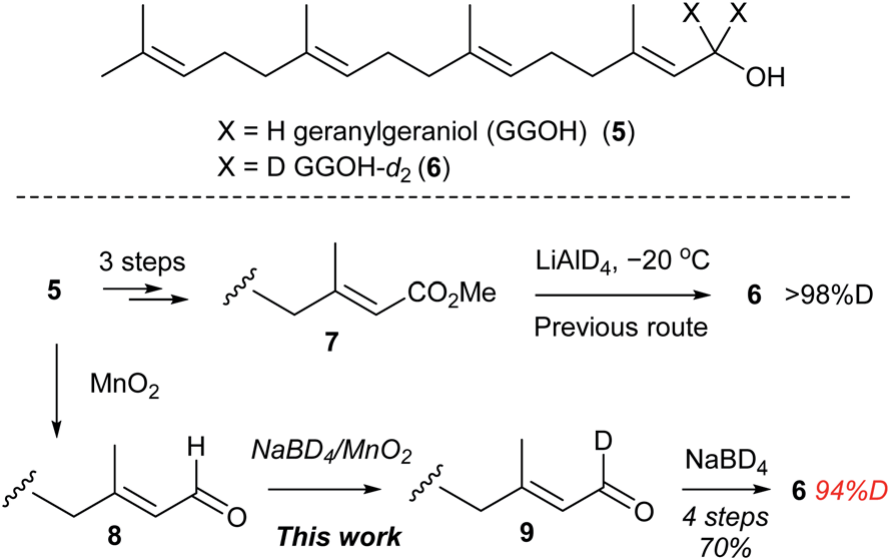
Synthesis of geranylgeraniol-*d*_2_ (6) from geranylgeraniol (5).

In conjunction with our recent efforts toward elucidation of biosynthetic reaction mechanisms of terpene synthases using d-labelled prenols,^15,16^ we needed geranylgeraniol-*d*_2_ (6) as an enzyme substrate. Previously, the synthesis of 6 (ref. 17) and other acyclic prenol-*d*_2_ derivatives^18^ was performed in four steps from 5*via* reduction of ester 7 with LiAlD_4_. However, commercially available LiAlD_4_ is almost out of stock in recently years. In addition, low temperature conditions (−20 °C) is required for the LiAlD_4_ reduction to avoid the undesired 1,4-reduction. We expected that NaBD_4_/MnO_2_ system would be an alternative to the LiAlD_4_ procedure to prepare 6, conveniently. According to the literature,^19^ geranylgeraniol (5) was converted to aldehyde 8 by MnO_2_ oxidation (Scheme 5). Aldehyde 8 was subjected to NaBD_4_/MnO_2_ to deliver d-enriched aldehyde 9 which was subsequently reduced by NaBD_4_ to provide geranylgeraniol-*d*_2_ (6) in 70% yield over four steps with satisfactory deuterium incorporation ratio (94% D). Under the conditions, the undesired 1,4-addition reaction was not observed. Thus, an operationally simple and mild deuteration of prenols-*d*_2_ was achieved by application of NaBD_4_/MnO_2_ system.

Previously, Brecker *et al.* investigated ^13^C kinetic isotope effects (KIEs) in the oxidation of cinnamyl alcohol using MnO_2_, Dess–Martin periodinane, and Swern oxidation (DMSO/(COCl)_2_/Et_3_N) to gain insight into the reaction mechanism.^20^ Comparison of the kinetic isotope of effects revealed the following order MnO_2_ > Dess–Martin oxidation ≈ Swern oxidation. The higher ^13^C KIE using MnO_2_ displayed that the C–H bond breaking in the intermediate is irreversible and rate-determining, and the oxidation proceeded *via* energy rich transition state. On the other hand, the lower ^13^C KIEs observed in Swern oxidation and Dess–Martin oxidation indicated that the intramolecular C–H bond cleavage in these oxidation reaction processes would not be slower to be rate-limiting.

Experimental results in Table 1 clearly shows that the degrees of %D are as follows MnO_2_ > PDC > TEMPO ≈ Dess–Martin > SO_3_–pyridine/DMSO. It is speculated that higher %D of MnO_2_ oxidation and lower %D of SO_3_–pyridine/DMSO oxidation would correlate to the ^13^C KIE data (MnO_2_ > Swern oxidation). It is interesting to note that the %D value in Scheme 4 depended on the substrates. The oxidation of propargyl alcohols 4w–4aa resulted in higher %D than those of the other alcohols. The oxidation of 4w–4aa needed a longer reaction period to complete the reactions. As mentioned in the previous ^13^C KIE studies, the rate limiting steps of the MnO_2_ oxidation relies on the C–H cleavage step of the reaction intermediate. It is considered that the slower C–H cleavage would provide the higher %D.

## 3. Conclusions

We have established a facile synthesis of aldehyde-*d*_1_ derivatives by NaBD_4_/MnO_2_ system. The new method is characterized by a high degree of functional group compatibility and a wide range of substrate scope including the synthesis of d-containing unsaturated aldehydes. Aromatic aldehyde-*d*_1_ derivatives such as 2c and 2g would be a useful synthetic intermediate for olefination, amination, hydride reduction, Suzuki cross coupling, and Sonogashira coupling reactions.^9*e*,10*c*^ Substituted acroleins and propynals can be used for Michael addition reaction, cycloaddition reaction, and transition metal catalysed transformations. In this context, NaBD_4_/MnO_2_ system would offer vital opportunity to the synthesis of highly functionalized d-labelled molecules *via* facile preparation of aromatic and unsaturated aldehyde-*d*_1_2. Deuterium-labelled compounds are often needed for the investigation of the mechanisms or determination of the rate-limiting step. The present synthetic method supports the studies from the viewpoint of the facile preparation of aldehyde-*d*_1_2 and its derivatives. Further application and mechanism studies are ongoing in our laboratory.

## Author contributions

Y. Yasuno and HO are contributed equally. Y. Yasuno, HO, and TS designed the synthetic route. TS wrote the manuscript. HO, Y. Yasuno, and AN prepared ESI.† HO, Y. Yasuno, AN, K. Kumadaki, K. Kitsuwa, KO, YT, and Y. Yamamoto performed syntheses of 2.

## Conflicts of interest

There are no conflicts to declare.

## Supplementary Material
